# Discovery biomarker to optimize obeticholic acid treatment for non-alcoholic fatty liver disease

**DOI:** 10.1186/s13062-023-00407-4

**Published:** 2023-08-25

**Authors:** Seung Min Lee, Dae Won Jun, Eileen Laurel Yoon, Ju Hee Oh, Yoon Jin Roh, Eun Jeoung Lee, Ji-Hee Shin, Young-Do Nam, Hyun Sung Kim

**Affiliations:** 1 Department of Translational Medicine, Graduate School of Biomedical Science & Engineering, Hanyang University, Seoul, Republic of Korea; 2 Department of Internal Medicine, Hanyang University Hospital, Hanyang University College of Medicine, 17 Haengdang-dong, Sungdong-gu, Seoul, 133-792 Republic of Korea; 3 Department of Obstetrics and Gynecology, Institute of Women’s Medical Life Science, Severance Hospital, Yonsei Cancer Center, Yonsei University College of Medicine, Seoul, Republic of Korea; 4 Department of Dermatology, Chung-Ang University Hospital, Seoul, Republic of Korea; 5 Research Group of Personalized Diet, Korea Food Research Institute, Wanju-gun, 55365 Republic of Korea; 6 Pathology, Medical genetic, Hanyang University College of Medicine, Seoul, Republic of Korea

**Keywords:** Non-alcoholic fatty liver, Obeticholic acid, Bile acid, Alternative pathway, Biomarker, Microbiome

## Abstract

The response rate to obeticholic acid (OCA), a potential therapeutic agent for non-alcoholic fatty liver disease, is limited. This study demonstrated that upregulation of the alternative bile acid synthesis pathway increases the OCA treatment response rate. The hepatic transcriptome and bile acid metabolite profile analyses revealed that the alternative bile acid synthesis pathway (Cyp7b1 and muricholic acid) in the OCA-responder group were upregulated compared with those in the OCA-non-responder group. Intestinal microbiome analysis also revealed that the abundances of *Bacteroidaceae, Parabacteroides*, and *Bacteroides*, which were positively correlated with the alternative bile acid synthesis pathway, were higher in the OCA-responder group than in the non-responder group. Pre-study hepatic mRNA levels of Cyp8b1 (classic pathway) were downregulated in the OCA-responder group. The OCA response rate increased up to 80% in cases with a hepatic Cyp7b1/Cyp8b1 ratio ≥ 5.0. Therefore, the OCA therapeutic response can be evaluated based on the Cyp7b1/Cyp8b1 ratio or the alternative/classic bile acid synthesis pathway activity.

**Supplementary Information:**

The online version contains supplementary material available at 10.1186/s13062-023-00407-4.

## Introduction

The prevalence of non-alcoholic fatty liver disease (NAFLD) is estimated to be as high as 25–30% [1]. However, no drugs have been approved for NAFLD therapy [2–5].

Bile acids (BAs) are synthesized by classical or alternative pathways, and 7α-hydroxylation of cholesterol is initiated primarily by cholesterol 7-α hydroxylase (CYP7A1) in the liver [6]. This is followed by oxidative cleavage of the side chain by steroid sterol 12-α hydroxylase (CYP8B1) [6, 7]. The alternative pathway is initiated by cholesterol 27-hydroxylase by sterol 27 hydroxylase (CYP27A1), and the oxysterol product is then hydroxylated by oxysterol 7-α hydroxylase (CYP7B1) [6, 8]. The classical pathway produces mainly Cholic acid (CA), while the alternative pathway produces Chenodeoxycholic acid (CDCA), which in rodents is mostly converted to muricholic acid (MCA) [9].

The alternative pathway catalyzes the first reaction by CYP27A1, but it is not the rate-limiting step in this pathway. An essential rate-limiting step of the alternative pathway is the transport of cholesterol to the inner mitochondrial membrane by the Liver X receptor (LXR; encoded by *NR1H3*) induced steroidogenic acute regulatory protein (StarD1) [10, 11].

BA synthesis is regulated to maintain homeostasis and prevent bile acid toxicity. BA synthesis has been reported to increase during the postprandial period and decrease during fasting in humans [12, 13]. Moreover, in previous studies, glucagon inhibited CYP7A1 gene expression and bile acid synthesis [14]. CYP7A1 gene expression increased dependent on the glucose concentration in HepG2 cells [15]. Retinoid-related orphan receptors (RORα encoded by *RORA*), which were important in lipid and glucose metabolism, positively regulate CYP7B1 by CYP7B1/RORE, but negatively regulates LXRα [16]. LXRα and RORα interact with each other and negatively regulate each other [16]. This study reveals a potential interaction between glucose signaling and bile acid synthesis.

Farnesoid X receptor (FXR; encoded by *NR1H4*), a transcription factor that is mainly expressed in the liver, intestine, and macrophages [17, 18], regulates the synthesis of BAs. Additionally, FXR regulates the expression of various genes involved in lipid metabolism [19]. Obeticholic acid (OCA, 6α-ethyl-chenodeoxycholic acid), an FXR agonist, is a potential therapeutic for NAFLD. A phase III clinical trial demonstrated that OCA alleviates hepatic fibrosis in 25% of patients [20]. Elafibranor (GFT-505) and selonsertib (ASK1 inhibitor) did not meet the primary endpoints in phase III clinical trials [21–23].

The unsatisfied results of most clinical trials on NAFLD can be attributed to the heterogeneity of patients with NAFLD who exhibit diverse pathophysiology even with similar phenotypes [24]. These various pathophysiology may vary depending on genetic background (e.g., *PNPLA3*), comorbidities (such as type 2 diabetes), and obesity [24–26]. Because of huge heterogeneity of NAFLD patients, it would be necessary to identify target population and biomarkers according to various NAFLD drugs for the prediction and/or monitoring the treatment response in NAFLD patients.

We aimed to identify an OCA-specific biomarker using a multi-omics-based approach to predict and monitor the treatment response in the NAFLD mouse model.

## Results

### Characteristics of the vehicle and OCA groups

**Fig. 1:**
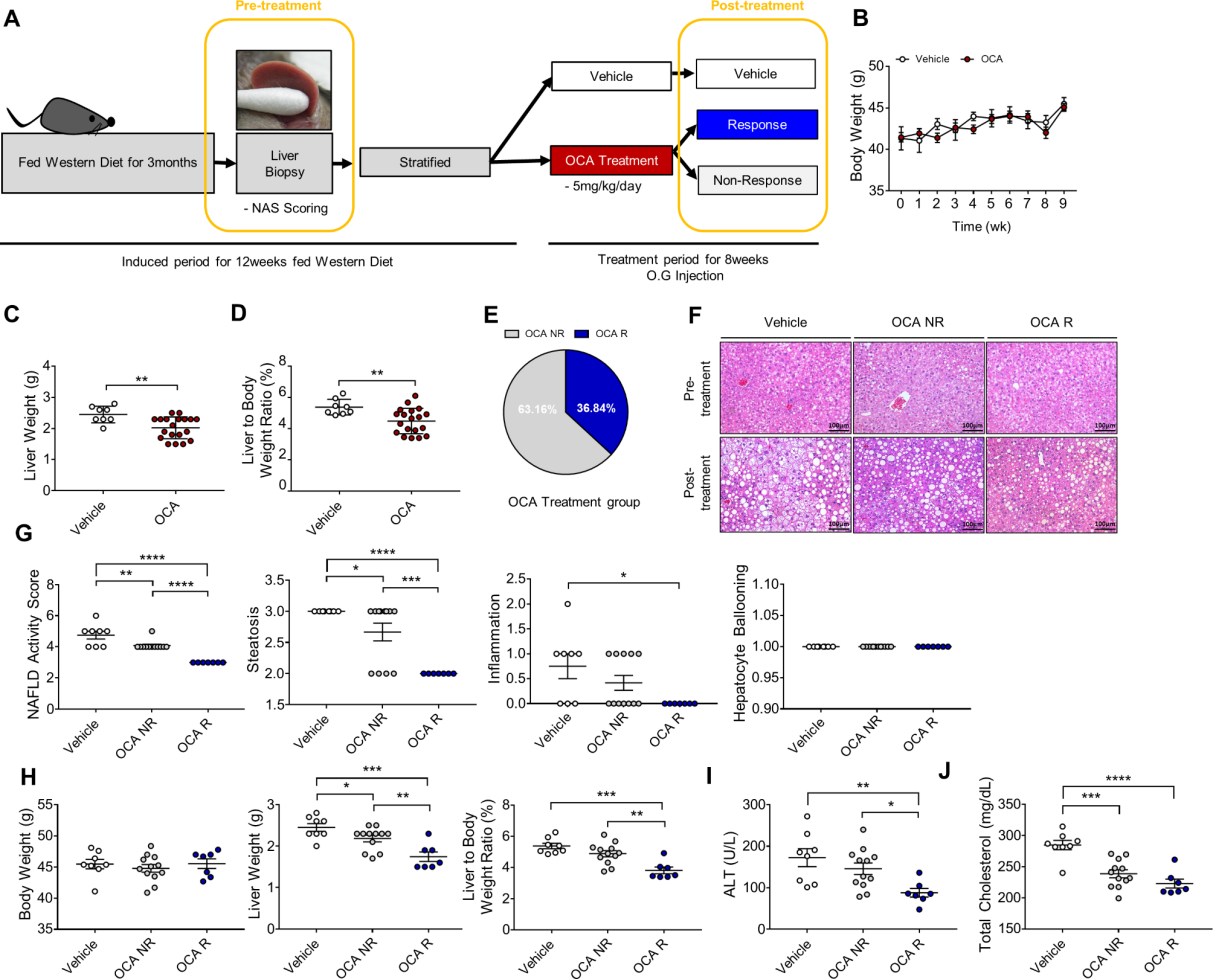
Obeticholic acid (OCA) response rates and characteristics of responder and non-responder in the non-alcoholic fatty liver disease (NAFLD) mouse model. (**A**) Schematic diagram of the study plan. (**B**) Average bodyweight of mice in the vehicle (n = 8) and OCA-treated groups (n = 19) during drug administration. (**C**) Liver weight and (**D**) liver-to-bodyweight ratio (%) of the NAFLD mouse model. Data are presented as mean ± standard error of mean. **p < 0.01 (Unpaired t-test). (**E**) Response rate in the OCA-treated group. (**F**) Hematoxylin and eosin staining of liver tissues at pre- and post-treatment. Scale bars, 100 μm. (**G**) Post-treatment NAFLD activity score. (**H**) Post-treatment bodyweight, liver weight, and liver-to-body weight ratio (%) of mice in the vehicle (n = 8), non-responder (n = 12), and responder (n = 7) groups. (**I**) Serum levels of alanine aminotransferase and (**J**) total cholesterol. Data are presented as mean ± standard error of mean. *p < 0.05, **p < 0.01, ***p < 0.001, and ****p < 0.0001 (one-way analysis of variance)

In total, 27 mice fed on a western diet were stratified into the vehicle (n = 8) and OCA groups (n = 19). The bodyweight and NAFLD activity score (NAS) were not significantly different between the two groups at baseline (Table 1). The liver weight and liver-to-bodyweight ratio in the OCA-treated group were significantly lower than those in the vehicle group at the end of treatment (Fig. 1B–D). Response rates of non-alcoholic steatohepatitis (NAS ≤ 3) of vehicle and OCA groups were 0% and 36.8%, respectively (Fig. 1E). The NAS, liver weight, liver-to-bodyweight ratio, and hepatic levels of alanine aminotransferase and total cholesterol in the OCA-responder group were significantly lower than those in the non-responder group (Fig. 1F–J).

**Table 1:** Baseline characteristics of mice

	Obeticholic acid (n = 19)	Vehicle (n = 8)	*p*-value
**Metabolic factors**			
Weight	41.5 ± 2.5	41.3 ± 4.0	0.92
**Liver histological findings**			
Total NAFLD activity score	4.2 ± 1.7	3.0 ± 1.6	0.35
Steatosis score	2.1 ± 0.8	1.5 ± 1.1	0.18
Lobular inflammation score	1.1 ± 1.1	0.5 ± 0.7	0.19
Hepatocellular ballooning score	1.0 ± 0.0	1.0 ± 0.0	1.00

Data are represented as mean ± standard deviation). NAFLD, non-alcoholic fatty liver disease

### Transcriptome analysis of the OCA-responder and non-responder groups

**Fig. 2:**
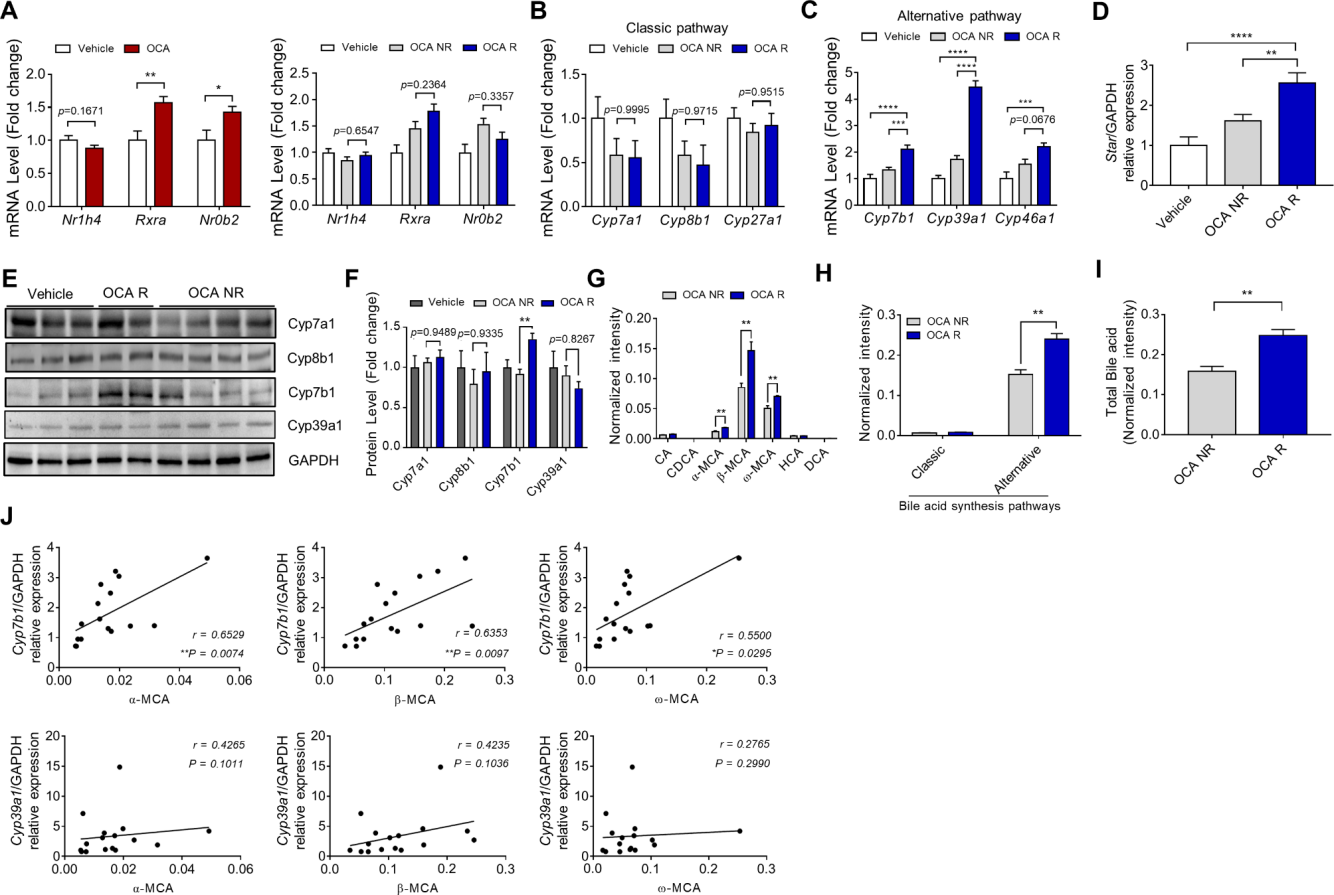
Comparative analysis of hepatic levels of obeticholic acid (OCA) target gene expression and bile acid composition in the responder and non-responder groups after treatment. (**A**) Comparative analysis of the hepatic levels of OCA target gene expression levels in the vehicle and OCA groups. Data are presented as mean ± standard error of mean. *p < 0.05, **p < 0.01, ***p < 0.001, and ****p < 0.0001 (Unpaired t-test and one-way analysis of variance). The expression levels of genes involved in the (**B**) classical and (**C**) alternative pathways of bile acid synthesis. (**D**) Comparative analysis of hepatic *Star* mRNA levels. Data are mean ± standard error of mean. **p < 0.01, ***p < 0.001, and ****p < 0.0001 (one-way analysis of variance). (**E**) Immunoblotting and (**F**) quantification of hepatic protein levels of Cyp7a1, Cyp8b1, Cyp7b1, and Cyp39a1 (vehicle (n = 8), non-responder (n = 11) and responder (n = 7)). Data are presented as mean ± standard error of mean. **p < 0.01 (one-way analysis of variance). (**G**, **H**, and **I**) Analysis of bile acid composition in the mouse liver tissue. Data are presented as mean ± standard error of mean. **p < 0.01 (Mann-Whitney U test). (**J**) Correlation between *Cyp39a1* and *Cyp7b1* mRNA levels and muricholic acids (MCAs). Correlation was analyzed using the nonparametric Spearman’s correlation coefficient. Differences were considered significant at *p <* 0.05

The hepatic transcriptome at the end of study was comparatively analyzed of the OCA-responder and non-responder between the vehicle and OCA groups. OCA target genes Nr1h4, which forms a heterodimer with Rxra, and Nr0b2, a target gene of Nr1h4, were compared and analyzed. *Nr1h4, Rxra* (*p* < 0.01) and *Nr0b2* (*P* < 0.05) mRNA levels were significantly upregulated in the OCA group but were not significantly different between the OCA-responder and non-responder groups (Fig. 2A). *Cyp7b1* (FC = 2.12; *p <* 0.0001) and *Cyp39a1* (FC = 4.46; *p <* 0.0001), which are involved in the alternative BA synthesis pathway, were significantly upregulated in the OCA-responder group compared with those in the vehicle and non-responder groups (Fig. 2B–C). Hepatic Star mRNA expression, which is a key rate limiting enzyme of alternative pathway, were significantly higher in the OCA-responder group than in vehicle and non-responder group (FC = 2.56; *p <* 0.0001) (Fig. 2D). Hepatic *Cyp7b1* protein levels in the OCA-responder group were significantly upregulated compared with those in the non-responder group (FC = 1.35; *p <* 0.01) (Fig. 2E–F).

### Hepatic bile acid compositions of the OCA-responder and non-responder groups

The levels of muricholic acids (MCAs) and metabolites of the alternative BA pathway were significantly upregulated in the OCA-responder group compared with those in the non-responder group (Fig. 2G–H). Additionally, the total BA level was higher in the OCA-responder group than that in the non-responder group (Fig. 2H–I). The levels of MCAs were significantly and positively correlated with those of Cyp7b1 (α-MCA, *r* = 0.65 and *P* = 0.007; β-MCA, *r* = 0.64 and *P* = 0.01; ω-MCA, *r* = 0.55 and *P* = 0.03) (Fig. 2J).

### Intestinal microbiome composition of the OCA-responder and non-responder groups

**Fig. 3:**
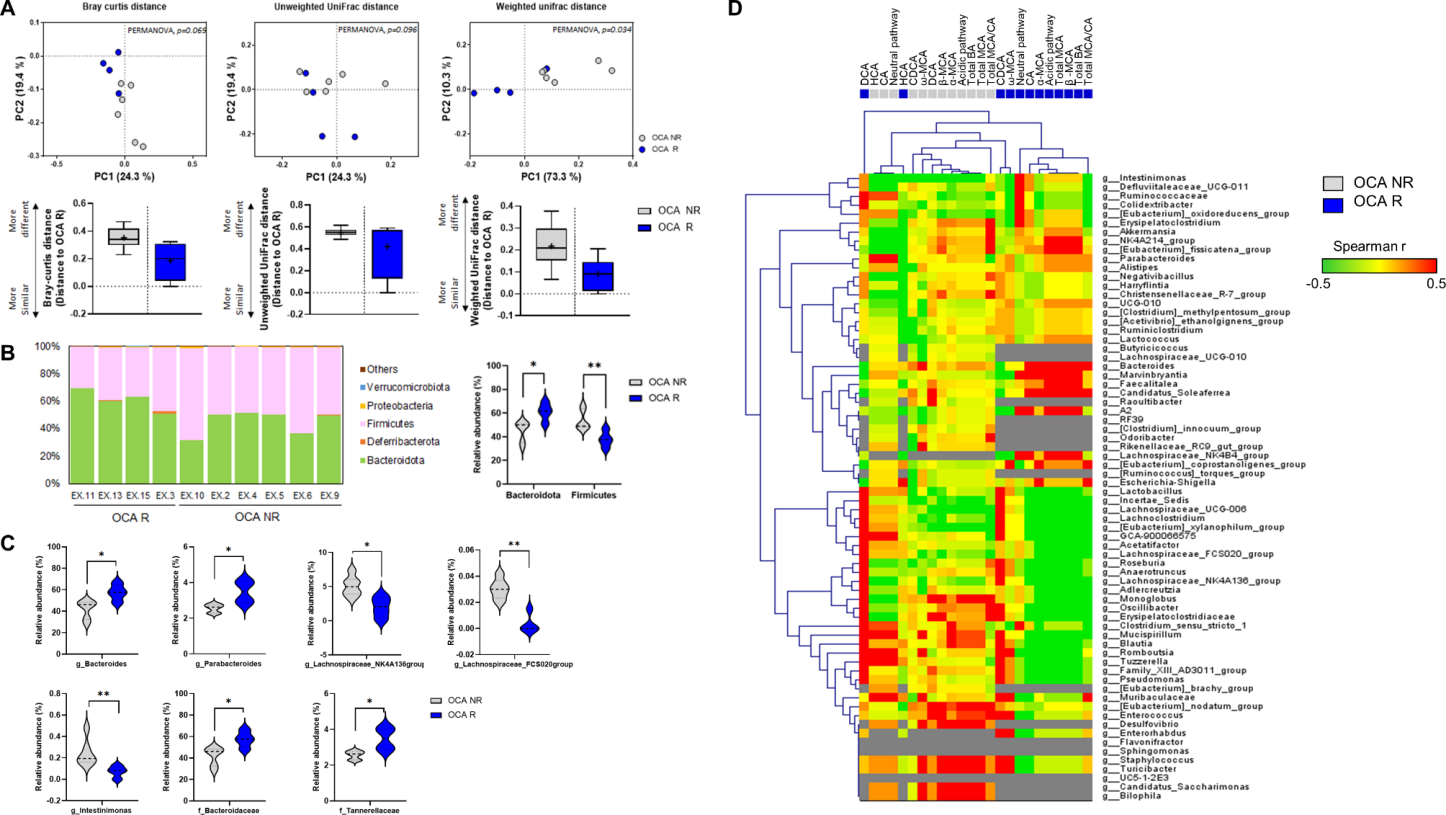
Comparison of microbiome structure between the responder and non-responder groups after treatment. (**A**) Principal-coordinate analysis of gut microbiota based on the Bray-Curtis, unweighted UniFrac, and weighted UniFrac distance. Significant P-values of PERMANOVA between groups emphasize the differences in microbial community structure. Grey and blue circles represent non-responders and responders, respectively. Box plots represent the median, lower and upper quartiles of the Bray-Curtis, unweighted UniFrac, and weighted UniFrac distances at each group comparing the effect of responsiveness of treatment on the community structure. Whiskers were calculated using the Tukey method. A lower distance indicates greater similarity compared to responders’ microbial communities. (**B**) Bar plots represent the relative abundances of phyla in responder (n = 4) and non-responder group (n = 6). Violin plots reporting the relative abundances of differentially abundant bacterial phyla between responder and non-responder group. Data are presented mean ± standard deviation. *p < 0.05 and **p < 0.01 (Mann-Whitney U test). (**C**) Violin plots reporting the relative abundances of differentially abundant bacterial genera between responders and non-responder group. Data are presented mean ± standard deviation. *p < 0.05 and **p < 0.01 (Mann-Whitney U test). (**D**) The heat map shows hierarchical clustering (Unweighted Pair Group Method with Arithmetic Mean, UPGMA method) of Spearman correlation coefficients between relative abundances of bacterial genera and metabolites in non-responders and responders samples using Euclidean distance. Positive and negative correlations are indicated by red and green colors, respectively, as shown in the color key. Missing values are indicated by grey spots

Abundance of microbial taxa in the OCA-responder and non-responder groups was comparatively analyzed (Fig. 3A, Fig. S1A, S1B). Abundances of *Firmicutes* were lower and the abundances of *Bacteroidetes* were higher in the OCA-responder group compared to those in the non-responder group (Fig. 3B, Fig. S1.C). The abundances of *Bacteroidaceae* and *Tannerellaceae* families and *Bacteroides* and *Parabacteroides* genera in the OCA-responders were higher than those in the non-responders. Meanwhile, the abundances of *Lachnospiraceae* family and *Intestinimonas* genus were significantly downregulated in the OCA-responder group (Fig. 3C). *Bacteroides* and *Parabacteroides*, which are reported to be positively correlated with the alternative BA synthesis pathway, were positively correlated with the OCA-responder group. Additionally, *Lachnospiraceae*, which is reported to be negatively correlated with the alternative BA synthesis pathway, was associated with the non-responder group (Fig. 3D).

### Post-study Cyp7b1/Cyp8b1 ratio of the OCA-responder and non-responder groups

We investigated the proportion of responders according to the expression of each gene involved in BA synthesis. After obtaining the average value of each gene expression, the proportion of responders for each gene was compared by dividing it into above-average and below-average.

**Fig. 4:**
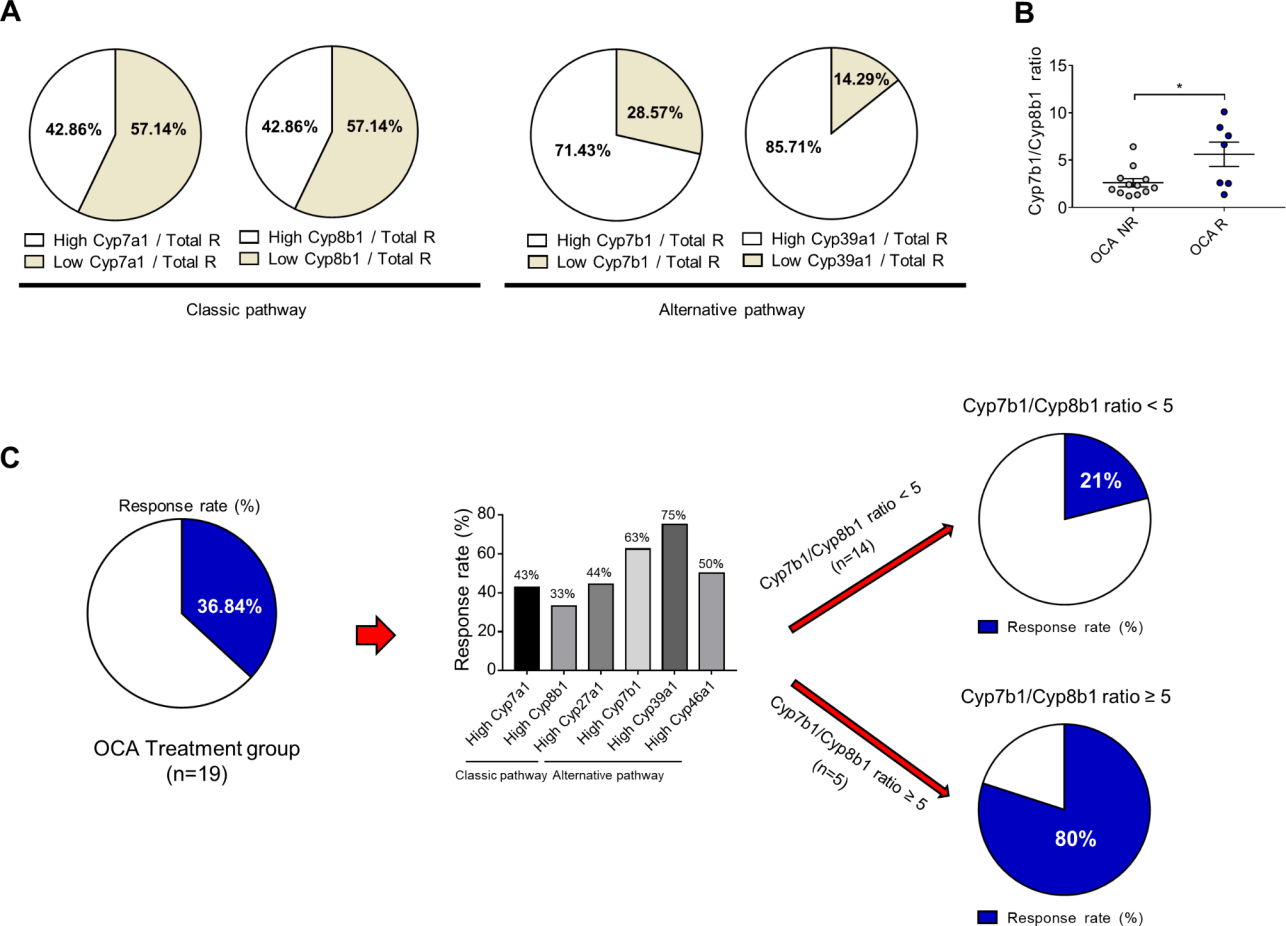
Responder classification based on the Cyp7b1/Cyp8b1 ratio. (**A**) The expression of bile acid-associated cytochrome P450 genes in the responder group relative to that in the OCA-treated mouse liver tissue. (**B**) Responder and non-responder classification according to the Cyp7b1/Cyp8b1 ratio. Data are presented as mean ± standard error of mean. *p < 0.05 (Unpaired t-test). (**C**) Ratio of responder mice among the OCA-treated mice overexpressing cytochrome P450 genes and the response rate according to the Cyp7b1/Cyp8b1 ratio

The hepatic Cyp7a1 and Cyp8b1 (classic BA synthesis pathway) levels were downregulated, whereas those of Cyp7b1 and Cyp39a1 (involved in alternative BA synthesis pathway) were upregulated in the OCA-responder group (Fig. 4A). The median Cyp7b1/Cyp8b1 ratio in the OCA-responder and non-responder groups were 5.6 and 2.6, respectively *(p <* 0.05) (Fig. 4B). The overall OCA response rate in the OCA group was 36.8%, which increased up to 80% in cases with a Cyp7b1/Cyp8b1 ratio ≥ 5.0 (Fig. 4C).

### Pre-study hepatic Cyp8b1 of the OCA-responder and non-responder groups

**Fig. 5:**
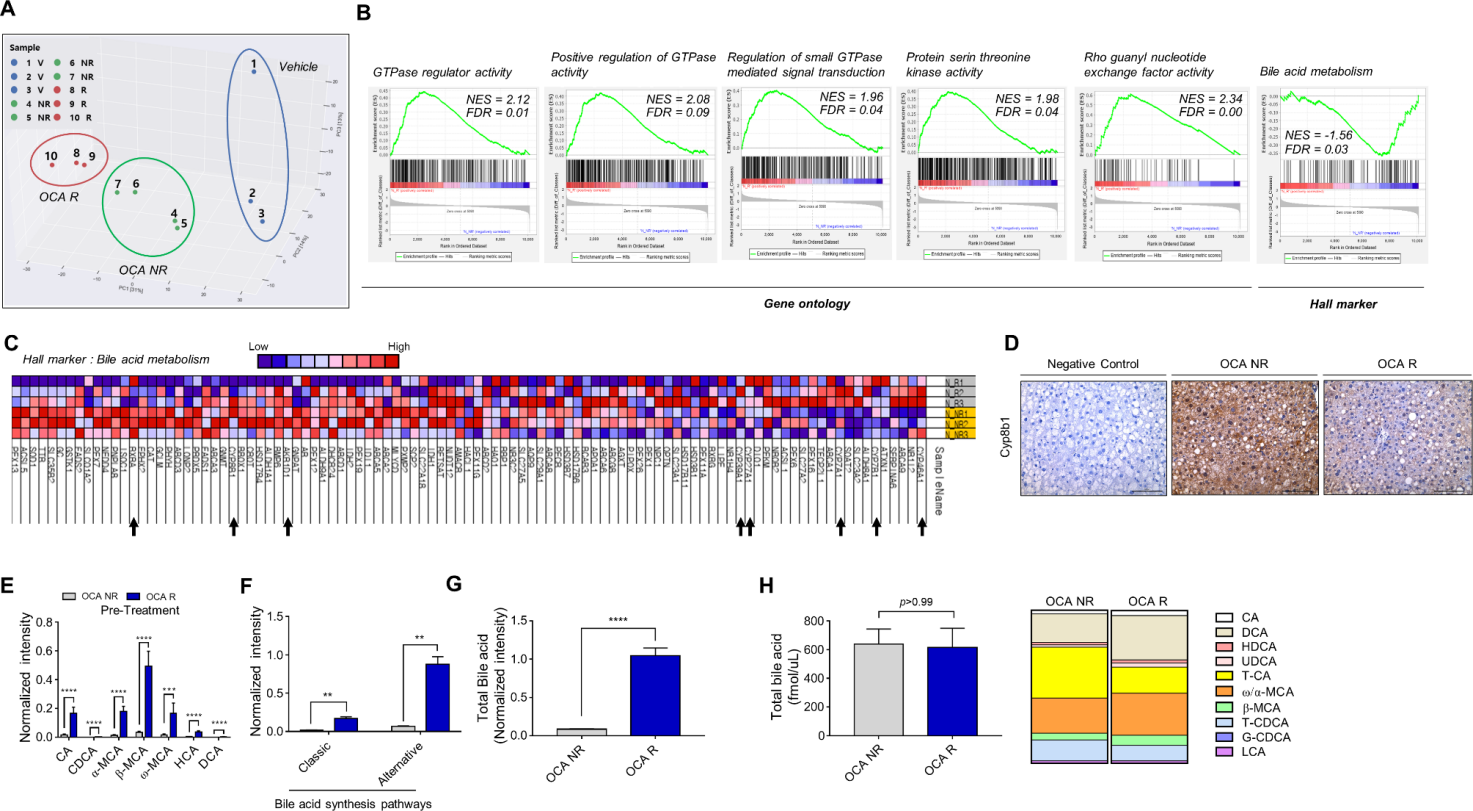
Multi-omics analysis of liver samples from the responder and non-responder groups before treatment. (**A**) Principal coordinate analysis plot and (**B**) gene set enrichment analysis (GSEA) results of the liver tissue transcriptome. (**C**) Heatmap of bile acid metabolism from GSEA. The hepatic levels of (**D**) Cyp8b1 (**E**, **F**, **G**) bile acids, and total bile acids. (**H**) Total bile acid and bile acid composition in the serum according to the response to OCA. Data are presented as mean ± standard error of mean. *p < 0.05, **p < 0.01, ***p < 0.001, and ****p < 0.0001 (Mann-Whitney U test) CA, cholic acid; CDCA, chenodeoxycholic acid; MCA, muricholic acid; HCA, hyocholic acid; DCA, deoxycholic acid; T-CA, taurocholic acid; LCA, lithocholic acid; T-CDCA, tauro chenodeoxycholic acid; G-CDCA, glycochenodeoxycholic acid; NES, normalized enrichment score; FDR, false discovery rate

The pre-study hepatic transcriptome at twelve weeks (baseline treatment) was comparatively analyzed according to OCA responder and non-responder (Fig. 5A–C). Pretreatment mRNA expressions of *Akr1d1*, *Cyp8b1*, and *Rxra*, which are surrogate markers for the classic BA synthesis pathway, were downregulated in the OCA-responder group compared to non-responder group (Fig. 5B–C). Additionally, pre-treated hepatic Cyp8b1 expression was lower in the OCA-responder group, (Fig. 5D). Hepatic levels of total BAs in the OCA-responder group were higher than those in the non-responder group (*p <* 0.0001) (Fig. 5E–G). Baseline serum MCAs concentration were higher and taurocholic acid (T-CA) was lower in the OCA-responder group compared to non-responder (Fig. 5E–H).

### Role of CYP7B1 in OCA treatment and it’s regulation

**Fig. 6:**
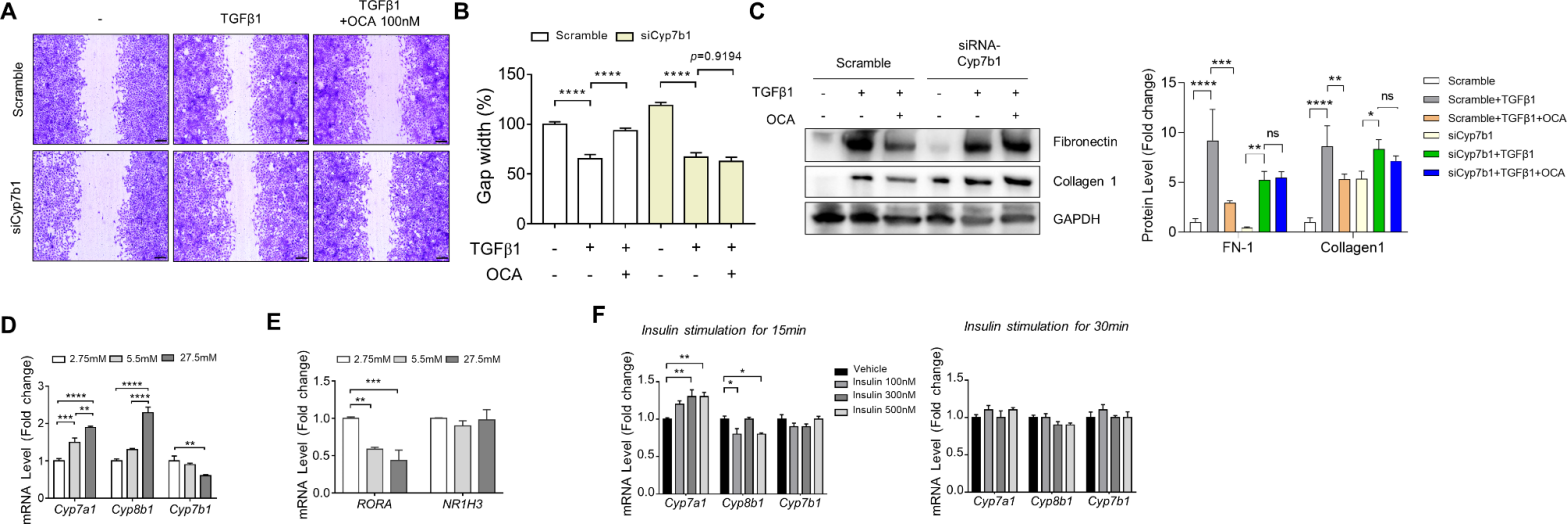
Effect of OCA on the LX-2 cells transfected with short-interfering RNA (siRNA) against***CYP7B1*(si-CYP7B1) and glucose on the expression levels of cytochrome P450 family genes related to bile acid synthesis.** (**A**) Wound healing assay using TGFβ1 and OCA in scramble and si-CYP7B1-transfected LX-2 cells. (**B**) A wound was introduced in the monolayer of scramble and si-CYP7B1-transfected cells. Wound healing was measured after 24 h. (**C**) Evaluation of fibrosis-related marker proteins. Comparative analysis of *CYP7A1*, *CYP8B1*, and *CYP7B1* mRNA levels according to (**D**) glucose concentrations in HepG2 cells. (**E**) *RORA* and *NR1H3* mRNA levels according to glucose concentration. Comparative analysis of *CYP7A1*, *CYP8B1*, and *CYP7B1* mRNA levels according to (**F**) insulin concentrations in HepG2 cells. Data are shown in bar diagrams as mean ± standard error of mean from three independent experiments. *p < 0.05, **p < 0.01, ***p < 0.001, and ****p < 0.0001 (one-way analysis of variance). Scale bars, 200 μm

OCA treatment inhibits the activation of LX-2 cells. Transfection with short-interfering RNA against *CYP7B1* (siCYP7B1) blocked the anti-fibrotic effect of OCA in LX-2 cells (Fig. 6A–C). OCA treatment downregulated the expression of TGFβ-induced fibronectin and collagen 1, but which was disappeared after transfection with siCYP7B1. Next, the effect of glucose on the mRNA levels of *CYP7A1*, *CYP8B1*, and *CYP7B1* was examined (Fig. 6D). Glucose upregulated the *CYP7A1* and *CYP8B1* mRNA levels and downregulated the *CYP7B1* mRNA levels in a dose-dependent manner (Fig. 6D). Additionally, glucose dose-dependently downregulated the *RORA* mRNA levels but did not affect the *NR1H3* mRNA levels (Fig. 6E). Furthermore, hepatic *CYP7A1*, *CYP8B1*, and *CYP7B1* mRNA levels were not dependent on insulin concentration (Fig. 6F).

## Discussion

This study demonstrated that the alternative BA synthesis pathway was upregulated, and the classical BA synthesis pathway was downregulated in the OCA-responder group compared with those in the non-responder group. In addition to transcriptome data, the results of BA proteome and intestinal microbiome analyses (as the abundance of *Parabacteroides* and *Bacteroides*) supported our hypothesis. The OCA response rate in the OCA-treated group was 37%, which increased up to 80% in case with a Cyp7b1/Cyp8b1 ratio of ≥ 5.0.

Strength of this study is that we used a histologically proven NAFLD mouse model. By performing pre-study liver biopsy to confirm the development of NAFLD, we could mimic the feature of human clinical trials as far as we can using mouse model. A high-fat diet may not always induce fatty liver in animals, and the degree of fatty liver induction varies between individual animals [9]. Therefore, liver biopsy before treatment could increase the reliability of animal study results. Moreover, pre-study liver biopsy can aid in narrowing down a drug-specific biomarker using multi-omic analysis to predict treatment responses. In this study, a drug-specific biomarker was identified between the OCA-responder and non-responder groups by multi-omics analysis of the liver biopsy before treatment.

Currently, drug-specific biomarkers to predict the treatment response in patients with NAFLD before treatment are not available. Identification of drug-specific biomarkers increases the success rate and decreases the sample size and the cost of clinical trials. Therefore, there is a need to identify a biomarker using a multi-omics approach for the prediction and monitoring of treatment responses in NAFLD.

The abundances of *Parabacteroides* were upregulated, whereas those of *Ruminococcaceae, Lanchospiraceae*, and *Eubacterium* were downregulated in the OCA-responder group. The intestinal microbiome can modulate bile composition. Conversely, bile composition can regulate the intestinal microbiome composition [9]. Gut microbes catalyze the epimerization of the 7-hydroxyl group of BA and activate 12α-dehydroxylase, which modulates the proportion of non-12-OH BAs [9, 27, 28]. Thus, the gut microbiota may regulate the expression of hepatic Cyp7b1. Previous studies have demonstrated that the alternative BA synthesis pathway was positively correlated with the abundances of *Parabacteroides, Mucispirillum, Bacteroides, and Prevotella* but was negatively correlated with the abundances of *Eubacterium, Clostridium*, and *Ruminococcaceae* [29, 30]. Further studies are needed to determine whether the abundance of *Parabacteroides*, *Bacteroidetes*, *Ruminococcaceae*, *Lanchospiraceae*, and *Eubacterium* can predict the OCA treatment response.

Our data showed that hepatic Cyp7B1 and Cyp39A1 expression correlated with OCA response. CYP7B1 and CYP39A1 are positively regulated by RORA [16]. The RORA response elements in the promoter and first intronic regions positively regulate CYP39A1 expression [31]. Additionally, RORA may be positively correlated with the alternative BA synthesis pathway. A recent study reported that RORA agonists attenuated fatty liver progression in NAFLD mice and regulate hepatic lipid homeostasis by modulating PPARγ [32, 33]. Consistently, RORA and CYP7B1 expression in HepG2 cells showed that glucose dose-dependently downregulated in this study.

This study showed that the OCA response depends on the CYP enzyme levels. It is very well known that CYP is an important enzyme for drug metabolism. There is possible that the cytochrome P450 system was involved in OCA drug metabolism. However, OCA is not metabolized by CYP enzymes in humans [34]. The cytochrome P450 enzyme CYP7B1 mediates the hydroxylation of carbons 6 and 7 of the B ring of oxysterol and steroids [35]. OCA has an alkyl group and a hydroxyl group at the corresponding position. Hence, the role of CYP7B1 in regulating the metabolism of OCA must be analyzed.

This study has several limitations. First, the expression of hepatic Cyp7b1 and activation of alternative BA synthesis pathways in the liver were discovered as biomarkers for the prediction of OCA treatment response in this study. However, hepatic Cyp7b1 or Cyp7b1/Cyp8b1 ratio expression was not quantitative and its invasive nature. It is necessary to discover a quantitative non-invasive biomarker that can evaluate the activity of hepatic Cyp7b1 or an alternative bile acid synthesis pathway in the liver. Our data showed that the abundances of *Bacteroides* and *Parabacteroides* were associated with the alternative BA synthesis pathways. Other study also suggested that abundances of Bacteroides and Parabacteroides associated with alternative BA synthesis pathways [9, 29, 30]. However, further studies are needed to determine whether the gut microbiome composition can be a predictive biomarker for OCA treatment response. Second, the roles of Cyp7b1 in the metabolism of OCA are unclear. Cyp7b1 modulates phase I drug responses, which may affect drug concentration. Hence, future studies needed the role of Cyp7b1 in OCA metabolism. In addition, our data seemed to be Cyp7b1 showed critical role of OCA action. siCYP7B1 blocked anti-fibrotic effect of OCA in LX-2 cells. Further evaluation using *Cyp7b1* knockout mice is needed in the future.

In conclusion, upregulated hepatic alternative bile acid synthesis pathway or high hepatic CYP7B1 can be a potential biomarker to monitor the OCA treatment response in NAFLD.

## Materials and methods

### Study design

After 12 weeks of feeding on a western diet, prior to treatment, pre-study liver biopsy was done to confirm the development of NAFLD (Fig. 1A). Biopsy proven NAFLD mice were randomly stratified into two groups (control and treatment groups). After 4 weeks, the mice were administered with either vehicle (control group) or OCA (5 mg/kg bodyweight/day, OCA group) through oral gavage for 8 weeks. The vehicle and OCA groups scarified at week 24 to evaluate the treatment response based on responder (NAS of ≤ 3) and non-responder.

### Animals

C57BL/6 mice obtained from Orient Bio (Seongnam, Korea) were maintained at a stable temperature (23 ± 2 °C) in a pathogen-free room under a 12-h light/dark cycle. To induce non-alcoholic fatty liver disease (NAFLD), mice were fed a western diet (D12079B, Research Diets Inc., NJ, USA) for 24 weeks. The mice were stratified based on the NAFLD activity score and randomly classified into the vehicle and obeticholic acid (OCA) groups.

### Pre-study liver biopsy in NAFLD animal model

Mice were anesthetized by intraperitoneal injection of zoletil® (tiletamine and zolazepam, 25 mg/kg) and Rompun® (xylazine hydrochloride, 25 mg/kg). The hair on the abdomen of the anesthetized mouse was shaved, and the incision area was sterilized with an iodine solution. Liver biopsy was performed after introducing a midline incision. To prevent bleeding, a heated spatula was applied to the biopsy site. The wound was closed with a 3 − 0 or 5 − 0 Vicryl suture [36]. The experimental protocol was approved by the Institutional Animal Care and Use Committee of Hanyang University (HY-IACUC-19-0030).

### Definition of OCA-responder and non-responder groups

To calculate the NAS, the mouse liver section stained with hematoxylin and eosin staining was scored. The OCA-responder group was defined as mice with an NAS of ≤ 3 at the end of treatment.

### Biochemical analysis

The blood samples in a serum separation tube (Vacutainer SST II Plastic serum tube, BD367955, Becton-Dickinson & Co., CA, USA) were centrifuged at 1500 rpm for 15 min. The serum was transferred to a new Eppendorf tube. Serum analysis was performed by an animal sample diagnosis company (KNOTUS. Co. Ltd., Guri, Korea). The serum levels of aspartate aminotransferase, alanine aminotransferase, total cholesterol, and triglycerides were analyzed using a Clinical analyzer 7180 (HITACHI, Japan).

### Histological and immunohistochemical analyses of the liver

Fresh liver tissues were fixed with 4% paraformaldehyde at 4 °C overnight and embedded in paraffin. The paraffin-embedded liver tissues were sectioned to a thickness of 4 μm and stained with hematoxylin and eosin solution. The sections were observed under a Virtual Microscope Axio Scan.Z1 (Zeiss, Oberkochen, Germany). Additionally, the liver sections were stained with anti-Cyp7b1 (MBS768409, MyBioSource, CA, USA) and anti-Cyp8b1 antibodies (PA5-37088, Thermo Fisher Scientific, MA, USA) overnight at 4 °C. After washing thrice with PBS containing 0.1% Tween-20 (PBS-T), the sections were incubated with the secondary antibodies in PBS for 30 min at 37 °C. The sections were washed thrice with PBS-T and incubated with an ABC reagent (PK-6100, Vector Lab, CA, USA) at room temperature for 30 min. Further, the sections were washed thrice with PBS-T, stained with 3,3′-diaminobenzidine (SK-4100, Vector Labs, CA, USA), dehydrated, and mounted. The sections were observed under a microscope (Virtual Microscope Axio Scan.Z1, Zeiss).

### Transcriptome analysis

Transcriptome analysis was performed by Macrogen (Seoul, Korea). Paired-end sequencing of the cDNA library (101 bp) was performed using a NovaSeq instrument. The sequence quality was confirmed with FastQC v. 0.11.7. The low-quality reads and adapter sequences were trimmed using Trimmomatic v 0.38 [37]. The preprocessed raw reads were aligned to the *Mus musculus* genome (mm10) using HISAT v2.1.0 [38]. In HISAT, two types of indices are used for alignment (a global, whole-genome index and tens of thousands of small local indexes), which were constructed using the same Burrows-Wheeler transform/graph Ferraggina-Manzini index as Bowtie2. Reference genome sequence and annotation data of *Mus musculus* (mm10) were downloaded from the UCSC table browser (http://genome.uscs.edu). Transcript assembly and abundance estimation were performed using StringTie [39, 40]. The relative abundance estimates were determined based on the fragments per kilobase of transcript per million mapped read (FPKM) values of the transcripts and genes. The FPKM values were used to comparatively analyze the gene abundance between samples.

### Transcriptome analysis of liver tissue

The relative gene abundance was expressed as fragments per kilobase of transcripts per million map reads (FPKM) using StringTie. One was added to all FPKM values of the filtered genes, except for genes with one or more zero-adjusted FPKM values in the sample, to facilitate log_2_ transformation. The filtered data were obtained after log_2_ transformation and quantile normalization. The differential gene expression data were analyzed using independent t-tests and expressed as fold changes (FCs). The statistical significance of differential expression data was determined using independent t-tests and FC, the null hypothesis of no difference between groups. The false discovery rate (FDR) was controlled by adjusting the *p*-value using the Benjamini-Hochberg algorithm. Differentially expressed gene sets were analyzed through hierarchical clustering with perfect connectivity and Euclidean distance as measures of similarity. Gene set enrichment analysis (GSEA v.4.03) was performed to determine whether a predefined set of genes was markedly enriched or depleted between the two phenotypes [41, 42]. The GSEA datasets were downloaded from http://www.gsea-msigdb.org/gsea/downloads.jsp.

### Metabolomic analysis of liver tissues

The liver tissue of BAs were analyzed at the EZ mass, Jeon Ju. Ultra-performance liquid chromatography quadrupole time-of-flight mass spectrometry and minimum reaction monitoring (MRM) data were collected, normalized, and aligned using UNIFI software version 1.8.2 (Waters). Data alignment was performed using retention time and mass windows of 0.2 min and 0.05 Da, respectively. The mass intensity of the target compound was normalized to the average mass intensity of the internal standard (terfenadien). BA metabolites were identified based on the MRM data and data from previous studies [43, 44]. Total BA contents were analyzed using the Mann-Whitney U test.

### Serum bile acid compositions

The serum levels of BAs were analyzed at the Asan Hospital, Seoul. Approximately 30 μL of mouse serum was preprocessed according to the internal standard manual and stored at − 20 °C. The samples stored at − 20 °C were reconstituted with 50% methanol and subjected to liquid chromatography-tandem mass spectrometry using a 1290 HPLC system (Agilent, Waldbronn, Germany) and QTRAP 5500 (AB Sciex, Toronto, Canada). MRM was performed in negative ion mode. The chromatography conditions were as follows: column temperature, 24 °C; flow rate, 200 μL/min. The ion chromatograms corresponding to specific transitions of each BA were used for quantification. The calibration concentration range for each metabolite was 0.1–10,000 nM (r^2^ ≥ 0.99). Data analysis was performed using Analyst 1.5.2.

### Intestinal microbiome analysis

Bacterial genomic DNA was extracted from fecal samples using the QIAamp DNA stool mini kit (Qiagen, Hilden, Germany), following the manufacturer’s instructions. The V1/V2 region of 16 S rRNA genes was amplified using an 8-base barcoded primer that tags the polymerase chain reaction (PCR) products of the samples. The amplicons were purified using a QIAquick PCR purification kit (Qiagen, Hilden, Germany). Next, the amplicons from different samples were pooled in equimolar concentrations. The quality and quantity of each constructed library were verified using a BioAnalyzer 2100 microfluidics-based device (Agilent, CA, USA). Sequencing was performed using an Ion Torrent Personal Genome Machine (Thermo Fisher Scientific, MA, USA), following the manufacturer’s instructions. The raw sequencing reads were analyzed using the QIIME 2 pipeline 29 (version 2020.11). Permutational multivariate analysis of variance was used to compare the differences in the gut microbial community among the groups. The abundance of microbial taxa between the two groups was analyzed using the Mann-Whitney U test. The p-values were corrected for multiple testing with the Benjamini-Hochberg correction (FDR). The basic statistical analyses were performed using GraphPad Prism, version 9.1.0 (GraphPad Software, Inc., CA, USA).

### Quantitative real-time PCR

Total RNA was extracted from the liver tissue and cells using TRIzol® reagent (15,596,026; Invitrogen, CA, USA). Quantitative real-time PCR was performed using a LightCycler®480 system (Roche Diagnostics, Mannheim, Germany) with LightCycler®480 SYBR Green I Master mix (Roche Diagnostics). The primers used for the analysis are listed in Supplementary Table 2.

### Western blotting

The liver tissues and cells were lysed in radioimmunoprecipitation assay lysis buffer containing a protease (P3100; Gene DEPOT, TX, USA) and phosphatase inhibitor cocktail solution (P3200; Gene DEPOT). The proteins in the lysates were subjected to SDS-PAGE. The resolved proteins were transferred to a 0.45 μm nitrocellulose membrane (GE Healthcare, Chicago, USA). The membrane was blocked with EzBlock Chemi (AE-1475, ATTO, Tokyo, Japan) and incubated with the primary antibodies overnight at 4 °C. The primers used for the analysis are listed in Supplementary Table 1. Next, the membrane was washed and incubated with horseradish peroxidase-conjugated anti-mouse, anti-rabbit antibodies (1:3000, GenDEPOT, TX, USA) at room temperature for 1 h. Immunoreactive signals were developed using Dyne ECL STAR (DN-250, Dyne Bio, Seongnam, Korea) and visualized using ChemiDoc™ (Bio-Rad, CA, USA).

### Cell culture

HepG2 cells (human liver cancer cells) were cultured at 37 °C in low-glucose Dulbecco’s modified Eagle’s medium (DMEM) containing 10% fetal bovine serum (FBS) and 1% penicillin-streptomycin (P/S). LX-2 cells (human hepatic stellate cell line) were cultured at 37 °C in high-glucose DMEM containing 2% FBS and 1% P/S. For glucose treatment, the cells were seeded and cultured under routine culture conditions for 24 h. The cells were then washed with 1× Dulbecco’s PBS (DPBS) and incubated with glucose/pyruvate-free DMEM (Gibco) containing 2.75, 5.5, or 27.5 mM glucose for 24 h.

### Wound healing assay

Wound healing assays were performed in 6-well tissue culture plates. LX-2 cells (2.8 × 10^5^ cells/well) cultured in 6-well plates for 24 h were transfected with short-interfering RNA (siRNA) for 4 h. A scratch was introduced in the monolayer, and the cells were incubated for 24 h. The culture medium was removed, and the cells were washed twice with 1× DPBS and fixed with 4% paraformaldehyde for 30 min. The cells were washed twice with 1× DPBS and stained with 1% crystal violet containing 2% ethanol for 30 min. After washing thrice with 1× DPBS, the cells suspended in DPBS were imaged using an optical microscope (Leica DM 400 B, Wetzlar, Germany).

### siRNA transfection

The cells were transfected with siRNA against human *CYP7B1* (Komabiotech, Seoul, Korea) using Lipofectamine RNAiMAX (Invitrogen), following the manufacturer’s instructions. Next, the cells were treated with TGFβ1 and OCA for 24 h. Scrambled siRNA was used as the negative control.

### Statistical analysis

All statistical analyses were performed using GraphPad Prism statistical software package (GraphPad Software, CA, USA). The means were analyzed using one-way analysis of variance or unpaired t-test. The data are represented as mean ± standard error of mean. Correlation analysis was performed with nonparametric Spearman’s correlation coefficient. Differences were considered significant at *p <* 0.05.

### Electronic supplementary material

Below is the link to the electronic supplementary material.

Supplementary Material 1

## Data Availability

mRNA sequencing data are publicly available in NCBI GEO via accession GSE215196.

## Abbreviations

CA: Cholic acid
CDCA: Chenodeoxycholic acid
DCA: Deoxycholic acid
FDR: False discovery rate
G-CDCA: Glycochenodeoxycholic acid
HCA: Hyocholic acid
LCA: Lithocholic acid
LXR: Liver X receptor
MCA: Muricholic acid
NAFLD: Non-alcoholic fatty liver disease
NAS: NAFLD activity score
NES: Normalized enrichment score
OCA: Obeticholic acid
StarD1: Steroidogenic acute regulatory protein
T-CA: Taurocholic acid
T-CDCA: Tauro chenodeoxycholic acid
