# Supplementary material for: Discovery biomarker to optimize obeticholic acid treatment for non-alcoholic fatty liver disease
Source: Biol Direct. 2023 Aug 25;18:50. doi: 10.1186/s13062-023-00407-4 (PMC10463927; doi:10.1186/s13062-023-00407-4)
Supplement: Supplementary file 1 — Supplementary Material 1 [file 13062_2023_407_MOESM1_ESM.docx]

**Supplementary Information**

**Discovery biomarker to optimize obeticholic acid treatment for non-alcoholic fatty liver disease**

Seung Min Lee^1^, Dae Won Jun^1,2*^, Eileen Laurel Yoon^2*^, Ju Hee Oh^1^, Eun Jeoung Lee^1^, Ji-Hee Shin^3^, Young-Do Nam^3^, and Hyun Sung Kim^4^

**Table of contents……………………………………………………………………………… 1**

**Supplementary Methods ……………………………………………………………….. 2 – 6**

**Supplementary Tables S1 – 2 ………………………………………………………….. 7 – 8**

Supplementary Table S1. Primary antibody list …………………………………………... 7

Supplementary Table S2. Oligonucleotide primer list for real-time PCR………..……… 8

**Supplementary Figures and Legends S1 …………………………………………… 9 – 10**

Supplementary Figure S1. Comparison of known microbiome with positive and negative correlations with alternative pathway between responder and non-responder groups after treatment………………………………………………………………………………... 9

Supplementary Figure S2. hepatic Nr1h3 and Rora gene expression in mice at the end of study……………………………………………………………………………………..... 10

**Supplementary methods**

*Animals*

C57BL/6 mice obtained from Orient Bio (Seongnam, Korea) were maintained at a stable temperature (23 ± 2 °C) in a pathogen-free room under a 12-h light/dark cycle. To induce non-alcoholic fatty liver disease (NAFLD), mice were fed a western diet (D12079B, Research Diets Inc., NJ, USA) for 24 weeks. The mice were stratified based on the NAFLD activity score and randomly classified into the vehicle and obeticholic acid (OCA) groups.

*Histological and immunohistochemical analyses of the liver*

Fresh liver tissues were fixed with 4% paraformaldehyde at 4 °C overnight and embedded in paraffin. The paraffin-embedded liver tissues were sectioned to a thickness of 4 µm and stained with hematoxylin and eosin solution. The sections were observed under a Virtual Microscope Axio Scan.Z1 (Zeiss, Oberkochen, Germany). Additionally, the liver sections were stained with anti-Cyp7b1 (MBS768409, MyBioSource, CA, USA) and anti-Cyp8b1 antibodies (PA5-37088, Thermo Fisher Scientific, MA, USA) overnight at 4 °C. After washing thrice with PBS containing 0.1% Tween-20 (PBS-T), the sections were incubated with the secondary antibodies in PBS for 30 min at 37 °C. The sections were washed thrice with PBS-T and incubated with an ABC reagent (PK-6100, Vector Lab, CA, USA) at room temperature for 30 min. Further, the sections were washed thrice with PBS-T, stained with 3,3′-diaminobenzidine (SK-4100, Vector Labs, CA, USA), dehydrated, and mounted. The sections were observed under a microscope (Virtual Microscope Axio Scan.Z1, Zeiss).

*Transcriptome analysis*

Transcriptome analysis was performed by Macrogen (Seoul, Korea). Paired-end sequencing of the cDNA library (101 bp) was performed using a NovaSeq instrument. The sequence quality was confirmed with FastQC v. 0.11.7. The low-quality reads and adapter sequences were trimmed using Trimmomatic v 0.38 [31]. The preprocessed raw reads were aligned to the *Mus musculus* genome (mm10) using HISAT v2.1.0 [32]. In HISAT, two types of indices are used for alignment (a global, whole-genome index and tens of thousands of small local indexes), which were constructed using the same Burrows-Wheeler transform/graph Ferraggina-Manzini index as Bowtie2. Reference genome sequence and annotation data of *Mus musculus* (mm10) were downloaded from the UCSC table browser (<http://genome.uscs.edu>). Transcript assembly and abundance estimation were performed using StringTie [33, 34]. The relative abundance estimates were determined based on the fragments per kilobase of transcript per million mapped read (FPKM) values of the transcripts and genes. The FPKM values were used to comparatively analyze the gene abundance between samples.

*Metabolomic analysis of liver tissue*

Lyophilized liver and fecal samples were extracted using a bullet blender (Next Advance, NY, USA). The metabolites in the supernatant obtained after centrifugation were analyzed using an ultra-performance quadrupole time-of-flight (UPLC-Q-TOF system) (Xevo G2-S; Waters, MA, USA). The extracts were injected into an Acquity UPLC BEH C18 column (2.1 mm × 100 mm; 1.7 mm; Waters). The chromatography conditions were as follows: elution time, 12 min; flow rate, 0.35 mL/min; column temperature, 40 °C; mobile phase, 0.1% formic acid gradient. The bile acid metabolites in the eluate were analyzed using a Q-TOF MS system in the positive multiple reaction monitoring mode. The MS conditions were as follows: scan time, 0.2 s; scan range, 50–1500 m/z; capillary voltage, 3 kV; sampling cone voltage, 40 V; desolvation gas flow rate, 800 L/h; temperature, 400 °C; source temperature, 100 °C. To ensure the accuracy of the mass measurement of metabolites, the leucine-enkephalin ([M + H] = 556.2771) standard was injected at a flow rate of 0.35 μL/min and a frequency of 10 s. Quality control samples were mixed with all samples and analyzed after every 10 analyses. The mass spectra were obtained using a collision energy lamp (10 to 30 eV). The MS data, including ionic strength, m/z, and retention time, were processed using UNIFI version 1.8.2 (Waters).

*Biochemical analysis*

The blood samples in a serum separation tube (Vacutainer SST II Plastic serum tube, BD367955, Becton-Dickinson & Co., CA, USA) were centrifuged at 1500 rpm for 15 min. The serum was transferred to a new Eppendorf tube. Serum analysis was performed by an animal sample diagnosis company (KNOTUS. Co. Ltd., Guri, Korea). The serum levels of aspartate aminotransferase, alanine aminotransferase, total cholesterol, and triglycerides were analyzed using a Clinical analyzer 7180 (HITACHI, Japan).

*Western blotting*

The liver tissues and cells were lysed in radioimmunoprecipitation assay lysis buffer containing a protease (P3100; Gene DEPOT, TX, USA) and phosphatase inhibitor cocktail solution (P3200; Gene DEPOT). The proteins in the lysates were subjected to SDS-PAGE. The resolved proteins were transferred to a 0.45μm nitrocellulose membrane (GE Healthcare, Chicago, USA). The membrane was blocked with EzBlock Chemi (AE-1475, ATTO, Tokyo, Japan) and incubated with the primary antibodies overnight at 4 °C. The primers used for the analysis are listed in Supplementary Table 1. Next, the membrane was washed and incubated with horseradish peroxidase-conjugated anti-mouse, anti-rabbit antibodies (1:3000, GenDEPOT, TX, USA) at room temperature for 1 h. Immunoreactive signals were developed using Dyne ECL STAR (DN-250, Dyne Bio, Seongnam, Korea) and visualized using ChemiDoc^TM^ (Bio-Rad, CA, USA).

*Quantitative real-time PCR*

Total RNA was extracted from the liver tissue and cells using TRIzol^®^ reagent (15596026; Invitrogen, CA, USA). Quantitative real-time PCR was performed using a LightCycler^®^480 system (Roche Diagnostics, Mannheim, Germany) with LightCycler^®^480 SYBR Green I Master mix (Roche Diagnostics). The primers used for the analysis are listed in Supplementary Table 2. The expression of target genes was normalized to that of *Gapdh* or *Actb* (internal control).

*Cell culture*

HepG2 cells (human liver cancer cells) were cultured at 37 °C in low-glucose Dulbecco’s modified Eagle’s medium (DMEM) containing 10% fetal bovine serum (FBS) and 1% penicillin-streptomycin (P/S). LX-2 cells (human hepatic stellate cell line) were cultured at 37 °C in high-glucose DMEM containing 2% FBS and 1% P/S. For glucose treatment, the cells were seeded and cultured under routine culture conditions for 24 h. The cells were then washed with 1× Dulbecco’s PBS (DPBS) and incubated with glucose/pyruvate-free DMEM (Gibco) containing 2.75, 5.5, or 27.5 mM glucose for 24 h.

*Wound healing assay*

Wound healing assays were performed in 6-well tissue culture plates. LX-2 cells (2.8 × 10^5^ cells/well) cultured in 6-well plates for 24 h were transfected with short-interfering RNA (siRNA) for 4 h. A scratch was introduced in the monolayer, and the cells were incubated for 24 h. The culture medium was removed, and the cells were washed twice with 1× DPBS and fixed with 4% paraformaldehyde for 30 min. The cells were washed twice with 1× DPBS and stained with 1% crystal violet containing 2% ethanol for 30 min. After washing thrice with 1× DPBS, the cells suspended in DPBS were imaged using an optical microscope (Leica DM 400 B, Wetzlar, Germany).

*siRNA transfection*

The cells were transfected with siRNA against human *CYP7B1* (Komabiotech, Seoul, Korea) using Lipofectamine RNAiMAX (Invitrogen), following the manufacturer's instructions. Next, the cells were treated with TGFβ1 and OCA for 24 h. Scrambled siRNA was used as the negative control.

**Supplementary table**

Supplementary table 1. Primary antibody list.

| Gene | Company | Product number |
| --- | --- | --- |
| Collagen1 | Abcam | ab138492 |
| CYP39A1 | Invitrogen | PA5-38909 |
| CYP7A1 | Bioss | bs-2399R |
| CYP7B1 | MyBioSource | MBS768409 |
| CYP8B1 | Invitrogen | PA5-37088 |
| Fibronectin | Abcam | ab2413 |
| GAPDH | Genetex | GTX100118 |

Supplementary table 2. Oligonucleotide primer list for real-time PCR.

| Gene | Forward Primer Sequences (5’ – 3’) | Reverse Primer Sequences (5’ – 3’) |
| --- | --- | --- |
| *Cyp27a1* | GAGAGTGAATCAGGGGACCA | TCAGGAATGGAGGGTTTCAG |
| *Cyp39a1* | TCATTCTGGAACCCTCCTGC | CAGCCCAAAGTACGACCAGT |
| *Cyp46a1* | CATGAGACTTCTGCCAACCA | CTTGGAACCGACAACCTCAT |
| *Cyp7a1* | CAACGGGTTGATTCCATACC | ATTTCCCCATCAGTTTGCAG |
| *Cyp7b1* | TTCTCTGGGCCTCTCTAGCA | CAGGGCTTCCATAGCTTCAG |
| *Cyp8b1* | TGGCCTCTTTCACTTCTGCT | CGGAACTTCCTGAACAGCTC |
| *Gapdh* | GTTGTCTCCTGCGACTTC | GGTGGTCCAGGGTTTCTT |
| *Nr0b2* | AGCTGGGTCCCAAGGAGTAT | GGTACCAGGGCTCCAAGACT |
| *Nr1h4* | TGGGTACCAGGGAGAGACTG | GTGAGCGCGTTGTAGTGGTA |
| *Nr2b1* | GCTCACCAAATGACCCTGTT | GTCTAGGGGCAGCTCAGAAA |
| *Star* | GGGCATACTCAACAACCAGGA | CGAAACACCTTGCCCACATC |
| *ACTB* | AGGAAGGAAGGCTGGAAGAG | AGAGCTACGAGCTGCCTGAC |
| *CYP7A1* | CCGATGGATGGAAATACCAC | GGCAGCGGTCTTTGAGTTAG |
| *CYP7B1* | CACCAGAGAACAATTGGACAGCC | GCTACCAAGTCTCCCTTTCGCA |
| *CYP8B1* | CTGGAGACCAAGCAGTCCTTTG | GATACTCCTGCCCACTGGACAT |
| *NR1F1* | CAGAGCAATGCCACCTACTCCT | CTGCTTGGACATCCGACCA |
| *NR1H3* | ATCGCCTTGCTGAAGACCTCTG | CTGCTTTGGCAAAGTCTTCCCG |

**Supplementary Figures**

**Figure S1. Comparison of known microbiome with positive and negative correlations with alternative pathway between responder and non-responder groups after treatment.** Comparative analysis of (A) microbiome known to be positively correlated with alternative pathways and (B) microbiome abundance known to be negatively correlated in responder and non-responder group. Data are presented as mean ± standard error of mean. ***P* < 0.01 (Mann-Whitney U test). (C) Firmicutes/Bacteroidota ratio of responder and non-responder group. Data are presented as mean ± standard error of mean. ***P* < 0.01 (Mann-Whitney U test).


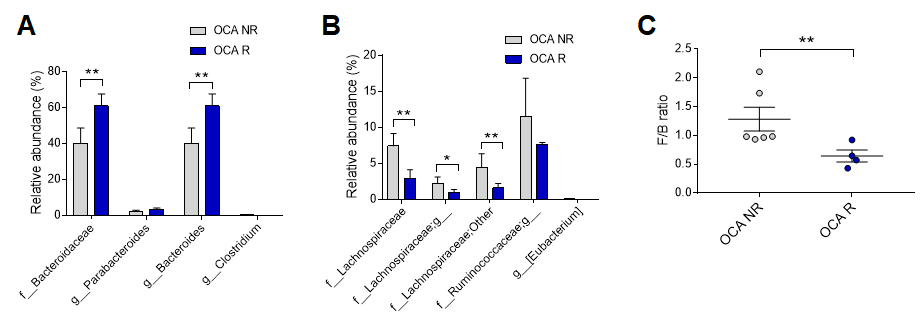


**Figure S2. hepatic Nr1h3 and Rora gene expression in mice at the end of study.** (A) Comparative analysis of hepatic *Nr1h3 and Rora* mRNA levels. Data are mean ± standard error of mean.

**
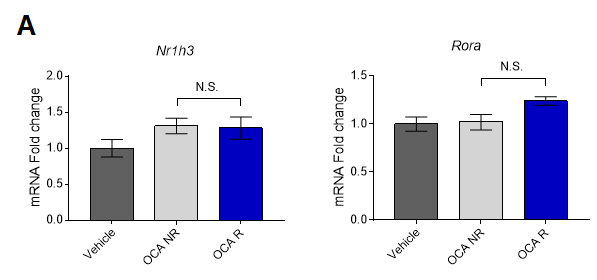
**
